# The role of MglA for adaptation to oxidative stress of *Francisella tularensis *LVS

**DOI:** 10.1186/1471-2180-12-14

**Published:** 2012-01-21

**Authors:** Marie Honn, Helena Lindgren, Anders Sjöstedt

**Affiliations:** 1 Department of Clinical Microbiology, Clinical Bacteriology, and Laboratory for Molecular Infection Medicine Sweden (MIMS), Umeå University, 90185 Umeå, Sweden

## Abstract

**Background:**

The *Francisella tularensis *protein MglA performs complex regulatory functions since it influences the expression of more than 100 genes and proteins in *F. tularensis*. Besides regulating the *igl *operon, it has been suggested that it also regulates several factors such as SspA, Hfq, CspC, and UspA, all important to stress adaptation. Therefore, it can be hypothesized that MglA plays an important role for *Francisella *stress responses in general and for the oxidative stress response specifically.

**Results:**

We investigated the oxidative stress response of the Δ*mglA *mutant of the live vaccine strain (LVS) of *F. tularensis *and found that it showed markedly diminished growth and contained more oxidized proteins than the parental LVS strain when grown in an aerobic milieu but not when grown microaerobically. Moreover, the Δ*mglA *mutant exhibited an increased catalase activity and reduced expression of the *fsl *operon and *feoB *in the aerobic milieu. The mutant was also found to be less susceptible to H_2_O_2_. The aberrant catalase activity and gene expression was partially normalized when the Δ*mglA *mutant was grown in a microaerobic milieu.

**Conclusions:**

Altogether the results show that the Δ*mglA *mutant exhibits all the hallmarks of a bacterium subjected to oxidative stress under aerobic conditions, indicating that MglA is required for normal adaptation of *F. tularensis *to oxidative stress and oxygen-rich environments.

## Background

*Francisella tularensis *is a facultative intracellular, gram-negative coccobacillus, which causes the potentially lethal disease tularemia. This zoonotic disease is transmitted via vectors such as ticks and mosquitoes and infects predominantly mammals such as small rodents, hares and rabbits [1]. The subspecies *tularensis *and *holarctica *also give rise to human infections. The pathogen is highly contagious, requiring as few as 10 bacteria to cause human infection, and subspecies *tularensis *causes a very aggressive disease with high mortality in humans if untreated [2]. The high virulence, ease of spread, and potentially high mortality of tularemia has led to the classification of *F. tularensis *as one of six category A select agents, *i.e*., the agents most likely to be used for bioterrorism [3]. In experimental infections, *F. novicida *and *F. tularensis *LVS are often used since both show significant virulence in small rodents but still are classified as BSL2 pathogens. The former species very rarely causes human infections and the latter is a human vaccine strain of subspecies *holarctica *origin [4].

An important virulence trait of *F. tularensis *is its ability to survive and multiply in an array of different cell types including hepatocytes and professional phagocytes [5]. The intracellular lifestyle relies on escape from the phagosome and the subsequent proliferation in the cytoplasm [6]. The mechanism of escape from the phagosome is not known but requires expression of the global regulator MglA (macrophage growth locus) [7]. This is most likely through its positive regulation of the genes belonging to the intracellular growth locus (*igl*) and other genes of the *Francisella *pathogenicity island. MglA together with an ortholog, SspA, forms a complex that directly interacts with the RNA polymerase [8] conferring a complex regulatory role that leads to the control of more than 100 genes and proteins in *F. tularensis *[9,10]. Besides the *igl *operon, it has been suggested that the activities of several stress-regulated factors, such as SspA, Hfq, CspC, and UspA, are linked to the MglA-dependent regulation [10]. Thereby, it plays an important role for the intracellular growth and stress responses in general and for the adaptation to oxidative stress response specifically.

Iron is essential for the survival of almost all living organisms. Limiting the amount of iron accessible to pathogens is therefore an important part of the host defence system [11]. Thus, it is essential for successful pathogens to circumvent this and they have evolved various strategies, such as the usage of siderophores, which are high affinity iron chelators synthesized in response to iron starvation [12]. Siderophore production in *Francisella *is dependent on proteins encoded in the *fsl *operon (Francisella siderophore locus) [13-15]. Besides the *fsl *operon, the ferrous iron transport protein FeoB may contribute to the iron sequestration in *F. tularensis*. Similar to most other genes related to iron uptake in bacteria, the *fsl *operon and *feoB *are under the negative control of Fur [[15,16]; Honn et al., unpublished]. When sufficient iron is available, Fur binds to a Fur box and thereby suppresses gene expression, whereas under low iron concentrations, Fur is released and transcription resumes. The iron uptake by the pathogens has to be fine-tuned since an excess of iron could be detrimental by potentiating the toxicity of H_2_O_2 _through the Fenton reaction, which generates highly reactive hydroxyl radicals and anions [17]. In fact, regulation of iron uptake, and oxidative stress are intimately linked, as evidenced by the regulation of iron uptake-related genes in, *e.g., Escherichia coli*. In this bacterium, *oxyR *is activated by H_2_O_2 _and causes an upregulation of Fur and catalase expression and this reduces the concentration of iron and H_2_O_2 _and thereby diminishes the Fenton reaction [18].

In the present study, we investigated how the Δ*mglA *mutant of LVS coped with oxidative stress. To this end, the accumulation of oxidized proteins in LVS and Δ*mglA *during growth was assessed and it was further tested if growth under microaerobic conditions affected oxidative stress parameters.

## Material and methods

### Bacterial strains

*Francisella tularensis *LVS, FSC155, was obtained from the American Type Culture Collection (ATCC 29684). The Δ*mglA *mutant of LVS has been described previously [7,19]. For complementation in *trans*, the intact *mglA *gene was amplified by PCR and cloned to pKK289Km [20], resulting in plasmid pKK289Km *mglA*. The resulting plasmid was then introduced into Δ*mglA *by cryotransformation and the resulting strain designated FUU301. The *katG *mutant has been previously described [21].

### Growth experiments

For liquid cultures, the *F. tularensis *strains were placed on McLeod agar plates (MC plates) that were incubated overnight under aerobic (20% O_2 _+ 0.05% CO_2_) or microaerobic condition (10% O_2 _+ 10% CO_2_) in an incubator with O_2 _+ CO_2 _control (Sanyo, Loughborough, UK). Bacteria from these plates were suspended in the Chamberlain's chemically defined medium (CDM), or in iron-depleted CDM (C-CDM), to an optical density at A_600 _nm (OD_600_) of ≈ 0.15. The latter media was used for depletion of the internal iron pool of the bacteria and was prepared as described previously [22]. The cultures were incubated overnight at 37°C and a rotation of 200 rpm under aerobic or microaerobic conditions. Thereafter, cultures were diluted in fresh CDM to an OD_600 _of 0.2 and cultivated as described above in the respective milieu. Iron-depleted bacteria were diluted in C-CDM to which 1,000 ng/ml FeSO4 had been added. Dilution and handling of the bacteria during the experiment were performed aerobically. Samples from these cultures were used to measure the levels of oxidized proteins, catalase activity, iron pool, gene expression and susceptibility to H_2_O_2 _of the bacteria.

For growth test on solid medium, the *F. tularensis *strains were richly streaked on MC plates that were incubated in 37°C and 5% CO_2 _over night. Bacteria were harvested, serially diluted in PBS and 100 μl of a dilution estimated to give approximately 100 colony forming units per plate were evenly spread on MC plates. The plates were incubated at 37°C in an aerobic or microaerobic milieu and the colony size scored after 2, 3, and 6 days of incubation.

### OxyBlot assay

The OxyBlot Protein Oxidation Detection Kit (Chemicon International) is based on a method for detection of carbonyl groups introduced into proteins by oxidative reactions. The carbonyl groups are derivatized to 2,4-dinitrophenylhydrazone (DNP-hydrazone) by use of 2,4-dinitrophenylhydrazine (DNPH) and can thereafter be detected by immunostaining. The OxyBlot kit was used to compare the amount of oxidized proteins in LVS and Δ*mglA *grown in an aerobic or a microaerobic milieu. Samples were collected at an OD_600 _of 0.6-0.7 and the bacteria were lysed using a buffer containing 2 M thiourea, 7 M urea, 4% CHAPS (3-[(3-Cholamidopropyl)dimethylammonio]-1-propanesulfonate), 0.5% ASB-14 (amidosulfobetaine-14), 1.0% DTT, 0.5 × protease inhibitor, and 1% β-mercaptoethanol. The amounts of protein in the samples were determined by use of the Bradford assay (Fermentas, St. Leon-Rot, Germany). The assay was carried out according to the manufacturer's protocol for Standard Bradford assay in microplates. Equal amounts of proteins were taken from each sample for derivatization and synthesis of negative controls according to the manufacturer's protocol. Briefly, samples were incubated with 1 × DNPH solution for 15 min at RT to allow derivatization of carbonyl-groups to DNP-hydrazone, after which a neutralization solution was added. Negative controls were prepared as the samples with the exception that they were treated with dH_2_O instead of 1 × DNPH solution, and therefore lack DNP-hydrazone. Negative controls were synthesized in order to ensure the specificity of the antibodies used for detection of DNP-moieties in oxidized proteins. Samples were blotted to PVDF membranes using a Bio-Dot Microfiltration Apparatus (BioRad), immunostained using a primary Rabbit anti-DNP antibody and a secondary Goat Anti-Rabbit IgG (HRP-conjugated) antibody; and developed with chemiluminescence to visualize the DNP-modifications, as directed by the instructions provided in the OxyBlot Kit. Samples were blotted at a concentration of 2.5 ng of protein in the first well followed by two-fold dilutions thereof.

### Catalase assay

LVS and Δ*mglA *were cultivated overnight in CDM and thereafter sub-cultured in CDM. When bacteria reached logarithmic growth phase (0.4-0.7 OD_600 _nm), the OD_600 _of the cultures were measured and 20-50 μl of culture was withdrawn and transferred to a 96-well UV-clear plate (Greiner Bio-One, Frickenhausen, Germany). To each well, PBS was added to give a final volume of 200 μl. Finally, 80 μl of 100 mM H_2_O_2 _in PBS was added to start the reaction. The decomposition of H_2_O_2 _was measured by monitoring the decrease in absorbance at 240 nm using a microplate reader (Paradigm, Beckman Coulter). Each strain was run in five replicates. The initial linear portion of the curve was used to calculate the Δ240 nm. A molar extinction coefficient of H_2_O_2 _at 240 nm of 43.6 M^-1 ^cm^-1 ^was used to calculated the concentration of H_2_O_2 _using the Beer-Lambert law, *A *= ε*cl*. One unit of catalase was defined as the amount that decomposes 1 μmol of H_2_O_2 _per minute per OD_600 _at 25°C.

### Analysis of gene expression

Bacteria were collected from cultures after 18 h of incubation and mixed with 50% (v/v) RNAlater (Qiagen, Hilden, Germany) and when needed, placed in -20°C, to stabilize the RNA until extraction could be performed. RNA was extracted using Trizol (Invitrogen) according to the manufacturer's protocol. cDNA was synthesized from this RNA and quantitative real-time PCR (RT-PCR) was used to analyze the cDNA samples. In order to remove contaminating DNA, the RNA samples were DNase-treated (DNA-*free *kit, Ambion, Inc, Austin, TX, USA) in accordance with the protocol supplied by the manufacturer. The RNA was quantified by Nanodrop (Thermo Fisher Scientific, Wilmington, DE, USA). cDNA was synthesized from 1 μg of the extracted RNA using iScript cDNA synthesis kit (Bio-Rad, Hemel, Hampstead, UK) according to the protocol provided by the manufacturer. To control for contaminating DNA in the RNA preparation, a control was prepared by substituting the enzyme from the cDNA synthesis for nuclease-free H_2_O (Ambion) (control 1). In order to degrade any remaining RNA, the cDNA was treated with 2.0 μl of 2.5 M NaOH at 42°C for 10 minutes after which the pH was adjusted by the addition of 5 μl of 1 M HCl. The samples were thereafter diluted and stored at -20°C.

RT-PCR was performed in the ABI Prism 7900HT Sequence Detection System (Applied Biosystems, Foster City, CA, USA) using the *Power *SYBR green PCR Master Mix (Applied Biosystems) as recommended by the manufacturer. Each reaction contained 12.5 μl of the SYBR green mix, 400 nM of forward and reverse primers, 5 μl of a cDNA and the total volume was adjusted with nuclease free water to 25 μl. Forward and reverse primers were obtained from Invitrogen and their sequences have been previously published [20,23] with the exception of the pairs used to measure *mglA*, *feoB *and *katG*. The sequences for *mglA *were the following: FTT1275-F, 5'-TTG CAG TGT ATA GGC TTA GTG TGA-3' and FTT1275-R, 5'-ATA TTC TTG CAT TAG CTC GCT GT-3', for *feoB*: FTT0249-F, 5'-TCA CAA GAA ATC ACA GCT AGT CAA-3' and FTT0249-R, 5'-CTA CAA TTT CAG CGA CAG CAT TAT-3' and for *katG *the following: FTT0721c-F, 5'-TTC AAG TTT AGC TGG TTC ATT CAT-3'and FTT0721c-R, 5'-GCT TGG GAT TCA GCT TCT ACT TAT-3'. The reactions were performed in MicroAmp 96-well plates (Applied Biosystems). The reactions were incubated at 50°C for 2 min, 10 min at 95°C followed by 45 cycles of 15 s at 95°C and 1 min at 60°C and a final cycle consisting of incubation at 95°C for 15 s, 60°C for 15 s, and at 95°C for 15 s. The lowest dilution that allowed detection of the gene within the linear working range was chosen as the dilution to be used for the analysis of the genes of interest. To control for contaminating DNA in the reaction, tubes with template from control 1 (see above) and tubes with water instead of template were included in the analysis. The controls gave Ct values (Ct is the threshold cycle) below detection level or at least 8 cycles later than the corresponding cDNA. Relative copy numbers (RCN) of selected genes were expressed in relation to the expression of the housekeeping gene *tul4 *[24] and calculated according to the following equation: RCN = 2^- ^Δ^Ct ^× 100 where ΔCt is Ct _(target) _- Ct_(*tul4*) _[25]. Thus, the copy number of a given gene is related to the copy number of *tul4*. Normalized Ct-values were used for statistical evaluation of the data.

### Chromazurol-S (CAS) plate assay

Chrome-azurol sulfonate-C-CDM agar plates (CAS plates) were prepared essentially as described [13]. Briefly, 40 ml of CAS/Fe(III)-hexadecyltrimethylammonium solution was mixed with 50 ml of a 4% (wt/vol) solution of GC II Agar Base (BD Diagnostic Systems, Franklin Lakes, NJ, USA) and 110 ml of C-CDM. The resulting CAS-C-CDM agar solution (1% agar) was poured into 20 ml Petri dishes. All components of the CAS-solution were purchased from Sigma-Aldrich, Buchs, Switzerland.

Bacteria were cultivated overnight in C-CDM and thereafter washed three times in C-CDM before dilution in C-CDM to 1.0 OD_600_. The suspension was added as a droplet of 2.5 μl to the center of the CAS plate. The plates were incubated at 37°C in 5% CO_2 _and the size and appearance of the halo formed around the bacterial colony was scored at 72 h.

### Ferrozine assay

A ferrozine-based method was used to measure the total amount of iron in the bacterial samples and in culture medium [26]. Ferrozine forms a complex with Fe^2+ ^that absorbs light at 562 nm. To determine the iron content of bacteria, a volume corresponding to 1.0 OD_600 _was withdrawn from the culture and bacteria collected by centrifugation for 5 min at 13,000 rpm. The bacteria were resuspended in PBS and collected by centrifugation. The resulting bacterial pellet was lysed with 100 μl of 50 mM NaOH. The solution was mixed thoroughly to ensure complete lysis of the bacteria. One hundred μl of 10 mM HCl was added to the lysate. To release protein-bound iron, the samples were treated with 100 μl of a freshly prepared solution of 0.7 M HCl and 2.25% (w/v) KMnO_4 _in H_2_O and incubated for 2 h at 60°C. All chemicals used were from Sigma-Aldrich. Thereafter, the samples were mixed with 100 μl of the iron detection reagent composed of 6.5 mM ferrozine, 6.5 mM neocuproine, 2.5 M ammonium acetate, and 1.0 M ascorbic acid dissolved in water. For determination of iron in medium, 30 μl of iron detection reagent was mixed with 170 μl of bacterial-free culture medium. The bacterial and medium samples were incubated with the iron-detection reagent for 30 min and insoluble particles were removed by centrifugation. Two hundred μl of the supernatant was transferred to a 96-well plate and the A_562 _determined in a microplate reader (Paradigm, Beckman Coulter, Bromma, Sweden). The iron content of the sample was calculated by comparing its absorbance to that of samples with FeCl_3 _concentrations in the range of 0-5,000 ng/ml that had been prepared identically to the test samples. The correlation coefficients of the standard curves varied between 0.998 and 0.999. The detection limit of the assay was 50 ng/ml Fe. The intra-sample variations (*i.e*., samples from the same culture) were less than 17 ng/OD_600_.

### H_2_O_2 _susceptibility test

Bacteria were cultivated overnight in CDM and thereafter cultured in fresh CDM for 2 h at 37°C and 200 rpm. The density of the cultures was measured and cultures were serially diluted in PBS to approximately 10^6 ^bacteria per ml. The exact number of bacteria at the start of the experiment was determined by viable count. The bacterial suspension was divided in 2 ml aliquots in 10 ml screw cap tubes. To some tubes H_2_O_2 _(Sigma) was supplied to reach a final concentration of 0.1 mM and other tubes were left untreated as controls. The tubes were incubated at 37°C 200 rpm. After 0 and 2 h of incubation, bacterial samples were collected and viable bacteria determined by plating 10-fold serial dilutions. The plates were incubated for 3 days at 37°C 5% CO_2 _before enumeration of the colony forming units (CFU).

### Statistical analysis

For statistical evaluation, two-tailed Student's *t*-test and two-tailed Pearson's correlation test in the statistical software program SPSS, version 16 were used.

## Results

### Growth of LVS and *ΔmglA *under aerobic or microaerobic conditions

**Figure 1:**
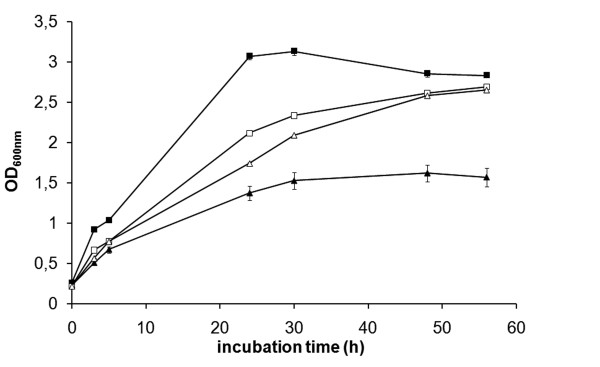
**Growth of LVS (squares) and Δ*mglA *(triangles) in CDM in an aerobic (closed symbols) or microaerobic (open symbols) milieu**. The diagram shows one representative experiment and similar results were seen in three additional experiments. The error bars represent the standard error of means and are included for all strains but are small for some data points and are therefore not visible in the diagram.

CDM is a liquid medium that effectively supports growth of *F. tularensis*. Accordingly, LVS grew to an OD_600 _of approximately 3.0 within 24 h under aerobic conditions, however, Δ*mglA *reached an OD_600 _of only slightly above 1.0 (Figure 1). In some experiments, LVS grew as well under microaerobic and aerobic conditions, but in other experiments, the growth was slightly reduced under the former condition (Figure 1). Δ*mglA *grew as well in the microaerobic as in the aerobic milieu during the first hours, but after approximately 24 h, its growth rate was reduced in the aerobic milieu, whereas it reached the same density as LVS in the microaerobic milieu after 48 h (Figure 1). FUU301 (Δ*mglA *expressing *mglA *in trans) exhibited an intermediary growth in the aerobic milieu and its density was 2.09 ± 0.05 *vs*. 2.59 ± 0.05 for LVS, whereas growth of the two strains was similar in the microaerobic milieu.

It was also tested if the growth of LVS and Δ*mglA *on solid medium was affected by the oxygen concentration. Approximately 100 bacteria were spread onto agar plates that were incubated in an aerobic or a microaerobic milieu. LVS formed colonies > two mm in size in both environments within 6 days but with delayed kinetics aerobically (Table 1). Δ*mglA *formed only few and small colonies on plates incubated aerobically. In the microaerobic milieu, however, it formed colonies of the same size as LVS, but with slightly delayed kinetics. Thus, regardless of growth medium used, Δ*mglA *appeared to exhibit markedly impaired growth under aerobic conditions.

**Table 1:** Size of colonies formed by LVS and Δ*mglA *on agar plates under aerobic or microaerobic conditions

	Colony size^a^			
	
Incubation time (days)	Aerobic		Microaerobic	
	
	LVS	Δ*mglA*	LVS	Δ*mglA*
2	0	0	1	0
3	1	0	2	1
6	3	MC^b^	3	3

^a ^Colony size was graded as follows: 0 = Not visible, 1 = colonies <1 mm in diameter, 2 = 1.0 -2.0 mm. 3 = >2 mm in diameter

^b ^Mixed colonies, a few large colonies growing in close proximity to each other but most colonies were hardly visible

### Oxidized proteins in LVS and Δ*mglA *cultivated under aerobic or microaerobic conditions

**Figure 2:**
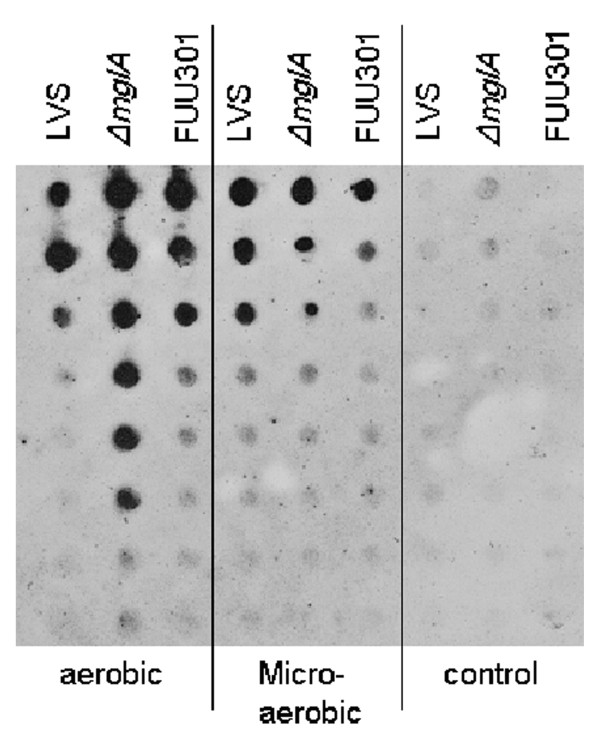
**Analysis of oxidized proteins by the Oxyblot assay**. Relative amounts of oxidized proteins in LVS, Δ*mglA*, or FUU301 during growth in an aerobic or microaerobic environment. Similar results were seen in two additional experiments. The first well of each preparation contained 2.5 ng of protein and the following wells two-fold dilutions thereof. Controls contain non-derivatized samples, and demonstrate the specificity of the antibodies used for detection of oxidative damage.

We hypothesized that the aberrant oxidative stress response of Δ*mglA *reported previously [8,10] may lead to suboptimal handling of the effects of oxidation. We therefore attempted to quantify such effects at a more general level. To this end, we analyzed the presence of oxidized proteins using the OxyBlot method. Preparations from Δ*mglA *cultivated under the aerobic conditions contained significantly more oxidized proteins than did those prepared from LVS (Figure 2). In contrast, the amounts of oxidized proteins were similar after cultivation in the microaerobic milieu. We noted some inter-experimental variation, but there were markedly increased amounts of oxidized proteins in the Δ*mglA *preparations under aerobic conditions in a majority of the experiments performed. FUU301 contained similar amounts of oxidized proteins as LVS regardless of growth condition (Figure 2).

In summary, the marked accumulation of oxidized proteins in Δ*mglA *during growth in the aerobic milieu strongly suggested that the mutant had an impaired response to oxidation. This may have been a reason for its delayed and lower maximal growth in the aerobic milieu.

### Catalase activity in LVS and Δ*mglA *cultivated under aerobic or microaerobic conditions

**Figure 3:**
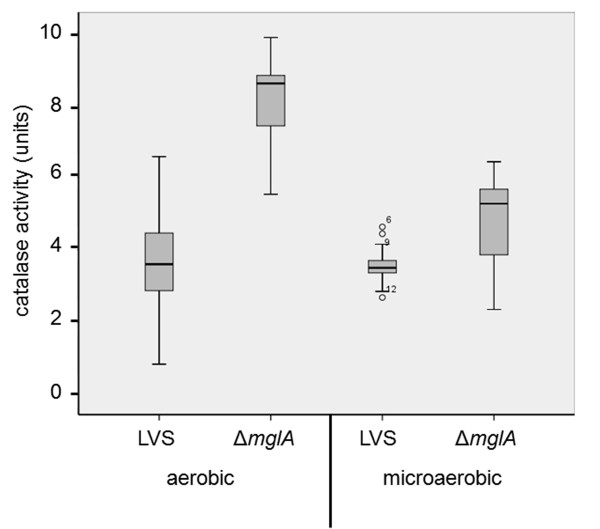
**Catalase activity of LVS and Δ*mglA***. Samples from cultures that were in the logarithmic growth phase were analyzed by the catalase assay. The line through each box shows the median, with quartiles at either end of each box. The T-bars that extend from the boxes are called inner fences. These extend to 1.5 times the height of the box or, if no case has a value in that range, to the minimum or maximum values. The points are outliers. These are defined as values that do not fall within the inner fences

As judged from the levels of oxidized proteins, Δ*mglA *experienced increased oxidative stress during growth in the aerobic milieu. *E. coli *responds to oxidative stress by upregulating the expression of catalase that degrades H_2_O_2 _and we asked if this was the case also for *F. tularensis *[18]. In addition, it has previously been demonstrated that the *F. novicida *Δ*mglA *mutant shows higher catalase activity than does the wild-type [10]. The catalase activity of LVS and Δ*mglA *was measured under aerobic and microaerobic conditions. The activity of LVS was similar under the two growth conditions, whereas Δ*mglA *showed significantly lower activity under microaerobic conditions (*P *< 0.001) (Figure 3). Still, Δ*mglA *demonstrated an elevated activity relative to LVS even under microaerobic conditions (*P *< 0.02) and even more so under aerobic conditions (*P *< 0.001) (Figure 3). An LVS *katG *deletion mutant did not decompose any H_2_O_2_, confirming that the experimental protocol is appropriate for measuring catalase activity.

In summary, the catalase activity of Δ*mglA *is strongly influenced by the oxygen concentration whereas no such correlation exists for LVS. This suggests that MglA is a factor that affects the regulation of the anti-oxidative response, particularly under aerobic conditions, and in its absence, the increased level of oxidation leads to a compensatory increase in the catalase activity.

### Regulation of the *fsl *operon by LVS and Δ*mglA*

Iron uptake is a factor that may be decreased by bacteria under oxidative stress in order to avoid toxic effects generated through the Fenton reaction [27]. Therefore, it would be logical if the iron regulation of Δ*mglA *is affected by the oxidative stress that occurs during aerobic growth. To assess this, we measured the expression of genes of the *fsl *operon and *feoB *by real-time PCR. Samples for the analysis were obtained after 18 h of growth, a time point when LVS had entered the stationary growth phase and the genes of the *fsl *operon were expected to be up-regulated due to iron deficiency.

In the aerobic milieu, LVS contained 4-12 fold more mRNA copies of *fslA-D*, 3.6-fold more copies of *feoB *(*P *< 0.001), and 2-fold less copies of *katG *than did Δ*mglA *(*P *< 0.05) (Table 2). Notably, *fslE *was not differentially regulated (Table 2). As expected, expression of *iglC *was greatly suppressed in Δ*mglA*. Importantly, the expression of all genes except for *katG *was restored to wild-type levels in the FUU301 strain when it was cultivated under aerobic conditions. FUU301 contained about 23-fold more mRNA copies of *mglA *than LVS. Notably, both LVS and FUU301 expressed significantly higher levels of *mglA *under microaerobic than aerobic conditions.

**Table 2:** Effect of growth condition on intra- and extra-cellular iron concentrations and gene regulation

Parameter tested	Growth condition					
	
	Aerobic			Microaerobic		
	
	LVS	Δ*mglA*	FUU301	LVS	Δ*mglA*	FUU301
Fe intra^a^	626 ± 27.2	661 ± 17.1	643 ± 24.5	893 ± 33.8	589 ± 21.9^d^	662 ± 20.5^d^
Fe extra^b^	B.D.L.^e^	186 ± 20.5	64.5 ± 8.97	73.9 ± 19.3	327 ± 10.7^d^	165 ± 46.1
Gene regulation^c^						
*fslA*	12.7 ± 0.64	2.51 ± 0.19^f^	10.6 ± 1.33	5.87 ± 0.71	4.93 ± 0.48	9.29 ± 1.19^g^
*fslB*	6.27 ± 0.39	0.83 ± 0.15^f^	5.6 ± 1.09	2.86 ± 0.43	1.87 ± 0.30	5.86 ± 0.30
*fslC*	5.96 ± 0.36	0.74 ± 0.15^f^	4.86 ± 0.68	2.61 ± 0.33	1.55 ± 0.28^g^	4.69 ± 0.26^g^
*fslD*	3.19 ± 0.23	0.97 ± 0.15^f^	3.52 ± 0.35	1.60 ± 0.23	2.40 ± 0.27^g^	3.73 ± 0.37^g^
*fslE*	0.82 ± 0.24	1.11 ± 0.15	1.55 ± 0.20^h^	1.04 ± 0.06	1.98 ± 0.14^d^	5.43 ± 1.20^d^
*feoB*	4.03 ± 0.29	1.37 ± 0.15^f^	4.95 ± 0.27	5.50 ± 0.41	4.33 ± 0.52	12.8 ± 3.77
*katG*	50.7 ± 8.62	110 ± 15.3^h^	116 ± 18.21^h^	79.1 ± 7.14	120 ± 19.3	135 ± 12.2i
*iglC*	390 ± 140	24.6 ± 5.37^f^	385 ± 58	685 ± 159	38.5 ± 15.9^d^	478 ± 120
*mglA*	16.5 ± 5.77	B.D.L.	384 ± 138^h^	63.7 ± 17	B.D.L.	637 ± 173^g^

^a ^The intracellular iron pool (ng/OD_600 _nm) of the strains after 18 h of growth

^b ^Iron (ng/ml) remaining in the culture medium after 18 h of growth

^c ^The expression of the genes was analyzed by quantitative real-time PCR. Results are expressed as RCN means ± SEM of results from four independent samples

^d ^*P *< 0.001 relative to LVS in the microaerobic condition

^e ^Below Detection Limit

^f ^*P *< 0.001 relative to LVS in the aerobic condition

^g ^*P *< 0.05 relative to LVS in the microaerobic condition

^h ^*P *< 0.05 relative to LVS in the aerobic condition

^i ^*P *< 0.01 relative to LVS in the microaerobic condition

Compared to the aerobic conditions, LVS down-regulated *fslA-D *2.5-fold under microaerobic conditions, whereas, in contrast, Δ*mglA *expressed 2-fold more of *fslA-D *microaerobically than aerobically. Overall, the adaptations under microaerobic conditions meant that *fslA-C *and *feoB *were expressed slightly higher and *fslD *and *fslE *almost 2-fold lower in LVS than Δ*mglA *(Table 2). The *fsl *genes were expressed at similar levels, and *feoB *was upregulated about 3-fold in FUU301 when cultivated in the microaerobic versus the aerobic milieu.

In summary, we observed that Δ*mglA *very markedly down-regulated the *fslA-D *and *feoB *genes compared to LVS under aerobic conditions but that differences were only marginal microaerobically, despite that less iron was present when Δ*mglA *had been cultivated under aerobic conditions. This supports our hypothesis that Δ*mglA *is subjected to oxidative stress under aerobic conditions and therefore needs to minimize iron uptake as a compensatory mechanism to avoid toxic effects of the Fenton reaction. Expression of *katG *was higher by the complemented FUU301 strain than by LVS under aerobic conditions, indicating that the former, as the Δ*mglA *mutant, may be experiencing a certain level of oxidative stress.

### Iron consumption and storage of LVS, Δ*mglA *and FUU301

The *fsl *genes and *feoB *are iron-regulated through Fur in *F. tularensis *[27]. Therefore, the expression of these genes may be a reflection of the iron content of the medium, or iron that is stored intracellularly and how these parameters correlate to each other. To assess this, these parameters were measured by the ferrozine assay. Importantly, the samples were obtained from the same cultures and time points as those analyzed by RT-PCR (Table 2).

The medium from aerobic and microaerobic Δ*mglA *cultures contained about 25% and 45%, respectively, of the iron initially supplied (735 ng/ml) (Table 2). This was significantly higher than for LVS cultures (*P *< 0.001 for both milieus). By use of Pearson's test it was found that for LVS there was no correlation between expression of *fslA-E *or *feoB *and the levels of iron remaining in the medium. For Δ*mglA*, medium from microaerobic cultures contained more iron than that from aerobic cultures (*P *< 0.001) (Table 2) and there was a correlation between the expression of *fslA *and *feoB *and the iron concentration of the medium (*P *< 0.05).

The iron pool of LVS was 1.4-fold higher in the microaerobic than in the aerobic milieu (*P *< 0.001) and there was a correlation between the expression of *fslA-D*, but not *fslE *and *feoB*, and the iron pool (*P *< 0.01). In contrast to LVS, the iron pool *of *Δ*mglA *did not increase under the microaerobic conditions and there was no correlation between the expression of *fslA-E *or *feoB *and the iron pool. The FUU301 strain was partly complemented for iron acquisition and storage (Table 2).

In summary, the intracellular iron pool but not the extracellular iron of LVS cultures strongly correlated to the regulation of the *fsl *operon. Thus, a low intracellular iron pool appears to be an important trigger of the expression of *fslA-D *in LVS. This correlation seemed not to exist in Δ*mglA *under aerobic conditions since Δ*mglA*, despite a low intracellular iron pool, had a repressed expression of *fslA-D *and *feoB*. The repressed expression of *fslA-D *and *feoB *was mitigated when Δ*mglA *grew under the microaerobic conditions, although extracellular iron levels were higher.

### Siderophore production and gene regulation by iron-starved LVS and Δ*mglA*

It was assessed if the suppressed expression of the *fsl*, *iglC*, and *feoB *genes in Δ*mglA *in the aerobic milieu occurred also if the strains were subjected to iron deficiency. To this end, LVS and Δ*mglA *were first cultivated in C-CDM to deplete their intracellular iron pool and thereafter cultured in C-CDM with 1,000 ng/ml of FeSO_4_. Under these conditions, expression of the *fsl *genes was similar in the two strains (Table 3).

**Table 3:** Gene regulation of iron-depleted LVS and Δ*mglA *grown under aerobic conditions

Gene	Gene regulation^a^	
	
	LVS	Δ*mglA*
*fslA*	31.2 ± 13.5	27.5 ± 10.5
*fslB*	3.75 ± 1.51	8.17 ± 4.03
*fslC*	3.22 ± 1.61	6.33 ± 3.83
*fslD*	1.33 ± 0.45	2.07 ± 0.87
*fslE*	0.27 ± 0.10	0.30 ± 0.13
*feoB*	0.37 ± 0.19	0.46 ± 0.27
*iglC*	428 ± 161	11.1 ± 5.41
*mglA*	19.2 ± 12.5	B.D.L.^b^

^a ^The expression of the genes was analyzed by quantitative real-time PCR. Results are expressed as RCN means ± SEM of results three to five independent samples

^b ^Below Detection Limit

The CAS plate assay is well-established for measurement of siderophore production in *F. tularensis *and we now used it to assess the siderophore production in Δ*mglA *[13,20,28]. We did not observe any significant difference between the mutant and LVS. However, it should be noted that minor differences with regard to the siderophore production may not be detected in the assay.

Together, the gene regulation of iron-starved bacteria and the CAS assay demonstrates that when subjected to severe iron-deficiency, Δ*mglA *regulates the *fsl *operon and similarly to LVS and has the capacity to produce siderophores. Thus, it appears to have no inherent defects with regard to iron uptake.

### Hydrogen peroxide susceptibility of LVS and Δ*mglA*

**Figure 4:**
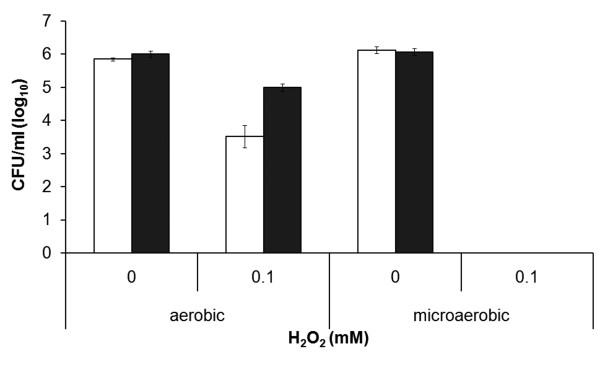
**Survival of LVS (white bars) or Δ*mglA *(black bars) after 2 h exposure to H_2_O_2 _Prior to the H_2_O_2 _challenge the bacteria had been cultivated for 2 h in CDM in the indicated milieu**. The bars represent the average from four experiments with triplicate samples of each. The error bars indicate the SEM

In view of the elevated catalase activity and aberrant iron uptake displayed by Δ*mglA*, we hypothesized that this would affect its susceptibility to H_2_O_2_. This was also the case since more than 2.0 log10 of LVS was killed during a 2 h incubation period when exposed to 0.1 mM H_2_O_2_, whereas the viability *of *Δ*mglA *decreased only 1.0 log10 by this treatment (*P *< 0.01) (Figure 4).

It was tested if growth in the microaerobic milieu, which diminished the catalase activity in Δ*mglA *and enhanced the iron uptake in LVS, affected the susceptibility of the strains to H_2_O_2_. Both LVS and Δ*mglA *were completely eradicated by a 2 h exposure to 0.1 mM H_2_O_2 _(Figure 4). In conclusion, our results show that the Δ*mglA *mutant compared to LVS displayed increased resistance to H_2_O_2 _under aerobic conditions whereas both showed markedly increased susceptibility to H_2_O_2 _under microaerobic conditions.

## Discussion

It is well established that MglA plays an important role for the intracellular growth and virulence of *F. tularensis*, most likely through its regulation of genes of the *igl *operon and other genes of the *Francisella *Pathogenicity Island. There are also reports that MglA regulates the oxidative stress response in *F. tularensis *[8,10] and that the *F. novicida mglA *mutant exhibits decreased survival during stationary-phase growth under nutrient-limiting conditions [10]. We observed that the LVS Δ*mglA *mutant did not grow to high densities in a nutrient-rich medium and generated only small colonies on solid agar plates. Here we asked how the *mglA *deletion mutant of LVS handled oxidative stress and if an impaired adaptation is the basis for its inability to grow to high densities.

The results of the Oxyblot assay showed that the Δ*mglA *mutant contained significantly more oxidized proteins than LVS under aerobic conditions. Reactive oxygen species are generated as a byproduct of the normal metabolism of a growing organism and there is, therefore, a continuous need to neutralize them to avoid oxidative damage of macromolecules in the cell. In view of this, the high level of oxidized proteins in Δ*mglA *strongly suggests that MglA has a central role for the normal oxidative stress response and that its absence renders *F. tularensis *severely impaired to handle reactive oxygen species leading to specific protein damage which hampers the bacterial growth. In support of this, previously published data on the *F. novicida mglA *mutant revealed that key enzymes in the glutaredoxin systems, such as gluthathione synthetase, glutaredoxine, and thioredoxine, all of which have critical roles to neutralize reactive oxygen species [29], were greatly repressed [9,10].

A rational adaptation to the increased oxidative stress encountered by Δ*mglA *would be to decrease the iron-driven Fenton reaction, which otherwise will result in the generation of highly reactive hydroxyl anions and radicals [17]. The most effective way to do this would be to limit the intracellular iron pool and upregulate the expression of catalase. Such an adaptation to oxidative stress has been noted in for example *E. coli *[18]. Our results support such a scenario also for *F. tularensis *since catalase was upregulated, thereby enhancing the capability of the bacterium to sustain an oxidative stress, and the expression of the *fsl *operon and *feoB *was suppressed in Δ*mglA *under aerobic conditions. Moreover, Δ*mglA *regulated iron-uptake genes similarly to LVS under microaerobic conditions and under severe iron-deprivation. This indicates that the marked downregulation of iron uptake genes observed under aerobic conditions does not relate to any inherent defects with regard to iron uptake, but instead is a compensatory mechanism needed to avoid the deleterious effects of the Fenton reaction.

An alternative explanation to the suppressed expression of the *fsl *operon and *feoB *in Δ*mglA *could be high availability of iron in the medium. However, we found no correlation between iron content and the *fsl *regulation, which further supports the hypothesis that oxidative stress was the primary reason for the dysregulation of the *fsl *operon and *feoB *in Δ*mglA *under aerobic conditions.

We hypothesized that the growth of Δ*mglA *in the microaerobic milieu would reduce the oxidative stress. Indeed, the levels of oxidized proteins in the Δ*mglA *mutant were normalized and similar to those found in LVS and, moreover, the growth of the mutant was similar to LVS. Other signs of reduced oxidative stress were the significantly reduced catalase activity and increased expression of the *fslA-D *and *feoB *genes. Collectively, all evidence indicates that MglA plays a critical role for the normal oxidative stress response and that its absence renders *F. tularensis *severely impaired to handle reactive oxygen species. We suggest that the lower levels of reactive oxygen species generated under growth in microaerobic conditions mitigated the defect of the mutant and, consequently, it grew as well as LVS under these conditions.

Our demonstration of an important role of MglA for the regulation of the *fsl *operon and catalase are in agreement with two previous publications [8,10], but if MglA directly regulates these genes is not known. Our present results suggest that the aberrant expression of catalase is an indirect effect of the increased oxidative stress of the Δ*mglA *mutant since the catalase activity was normalized under the microaerobic conditions. Similarly, the mutant normalized expression of *fslA-D *and *feoB *under the microaerobic conditions and this also occurred under severe iron deficiency. In contrast, *iglC*, known to be transcriptionally regulated by MglA, was repressed in Δ*mglA *regardless of growth conditions or iron availability. Together these data imply that there are also MglA-independent mechanisms that transcriptionally regulate the *fsl*, *feoB *and *katG *genes in *F. tularensis.*

The increased catalase activity in the Δ*mglA *mutant is a likely explanation for the high resistance of the mutant to H_2_O_2_. Such a correlation was also reported for *F. novicida *[10]. Besides catalase, the size of the intracellular iron pool is a factor that determines the susceptibility of *F. tularensis *to H_2_O_2 _[22]. We recently showed that subspecies *holarctica *strains, including LVS, contain more iron and were more susceptible to H_2_O_2 _than strains of subspecies *tularensis *[22]. When the iron pool of the subspecies *holarctica *strains was depleted, their susceptibility to H_2_O_2 _decreased. Here we observed that LVS sequestered significantly more iron under the microaerobic conditions. Since iron is a factor that determines the susceptibility of *F. tularensis *to H_2_O_2_, it is very likely that the substantial iron pool of LVS under the microaerobic conditions contributed to its extreme susceptibility to H_2_O_2_. Iron could, however, not explain the high susceptibility of Δ*mglA *to H_2_O_2 _in the microaerobic milieu, but in this case the decreased activity of catalase is a probable explanation for its reduced ability to handle the toxic effects. This agrees with our previous findings that catalase plays a very important role for LVS in protection against H_2_O_2 _[21].

The present study confirms previous findings that MglA plays an important role for the adaptation to oxidative stress in *F. tularensis *LVS and, moreover, we demonstrate that the role of MglA is most critical during growth in an aerobic milieu, whereas its importance is less obvious in an oxygen-restricted milieu. Therefore, we hypothesize that MglA is of special importance for the bacterium to survive in oxygen-rich foci.

## Conclusions

We made the important observation that a major factor for the diminished growth of Δ*mglA *appeared to be its impaired adaptation to a normal oxygen environment since its growth was normalized under microaerobic conditions. The growth defect of the mutant reflects the important role of MglA for the antioxidant defense and the data show there are MglA-independent mechanisms that transcriptionally regulate the *fsl *operon, *feoB*, or *katG*. In addition, our data indicate that LVS copes with oxidative stress by concomitantly upregulating detoxifying enzymes and downregulating iron sequestration.

## Correspondence

Anders Sjöstedt, Department of Clinical Microbiology, Umeå University, SE-901 85 Umeå

## Competing interests

The authors declare that they have no competing interests.

## Authors' contributions

MH carried out the growth experiments, OxyBlot assay, gene expression studies, CAS-plate assay, H_2_O_2 _susceptibility test, participated in the design of experiments, analysis of collected data and drafting of the manuscript. HL carried out the catalase assay, ferrozine assay and statistical analysis, conceived of, and designed the experiments, analyzed the collected data and drafted the manuscript. AS conceived of the study, participated in its design and coordination, and drafted the manuscript. All authors read and approved the final manuscript.
